# Effects of turning frequency on fermentation efficiency and microbial community metabolic function of sheep manure composting on the Qinghai–Tibet Plateau

**DOI:** 10.1186/s40643-023-00675-y

**Published:** 2023-08-21

**Authors:** Rui Cai, Sasa Zuo, Xiaohui Cao, Xin Jiang, Chuncheng Xu

**Affiliations:** College of Engineering, China Agricultural University, No. 17 Qinghua Donglu, Haidian District, Beijing, 100083 China

**Keywords:** Sheep manure composting, Qinghai–Tibet Plateau, Turning frequency, Fermentation efficiency, Microbial community metabolic function

## Abstract

This study explored the effects of turning frequency on fermentation efficiency and microbial metabolic function of sheep manure composting on the Qinghai–Tibet Plateau (QTP). Five treatments with different turning frequencies were set up in this study: turning every 1 day (T1), 2 days (T2), 4 days (T3), 6 days (T4), and 8 days (T5). Results showed that the high temperature period for T1 and T5 lasted only 4 days, while that for T2–T4 lasted more than 8 days. The germination index of T1 and T5 was lower than 80%, while that of T2–T4 was 100.6%, 97.8%, and 88.6%, respectively. This study further predicted the microbial metabolic function of T2–T4 using the bioinformatics tool PICRUSt2 (Phylogenetic Investigation of Communities by Reconstruction of Unobserved States) and determining the activities of various functional enzymes. The results showed that carbohydrate metabolism, protein metabolism, and nucleotide metabolism were the main metabolic pathways of microorganisms, and that T2 increased the abundance of functional genes of these metabolic pathways. The activities of protease, cellulase, and peroxidase in T2 and T3 were higher than those in T4, and the effect of T2 was more significant. In conclusion, turning once every 2 days can improve the quality of sheep manure compost on the QTP.

**Graphical Abstract:**

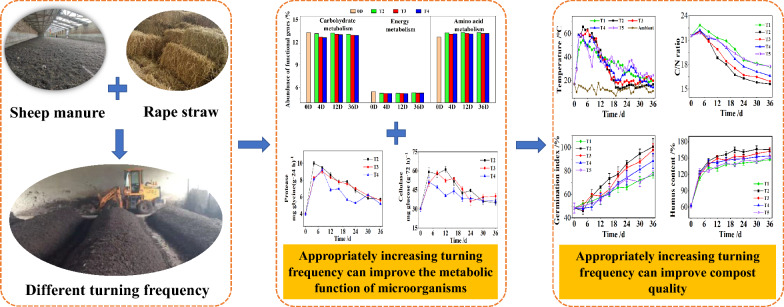

## Introduction

The Qinghai–Tibet Plateau (QTP) is the most important sheep breeding area in China. At the end of 2021, it had a population of more than 23 million sheep; these sheep generate and accumulate large amounts of manure, especially on large ranches (Cai et al. 2023a). Sheep manure has become a potent source of pathogens and antibiotics that can lead to soil and water pollution around ranches and pose a threat to staff and animal health (Yu et al. 2018; Ravindran et al. 2019). The efficient disposal and use of this waste is beneficial for the ecological environment and sustainable development of these ranches.

Aerobic composting is an efficient method for mitigating environmental damage and ensuring effective resource use for livestock and poultry waste (Chen et al. 2020). However, the QTP is characterized by a harsh climate with extreme cold and hypoxic conditions (Guo et al. 2021). This mean that the oxygen content of the composting pile there is lower than that of other regions. An extremely low oxygen content can severely affect the growth and metabolism of aerobic microorganisms and promote the production of malodorous gases and greenhouse gases by anaerobic microorganisms, which then leads to environmental pollution (Iqbal et al. 2010; Guo et al. 2012; Li et al. 2013). Therefore, oxygen supplementation is of great significance to compost production on the QTP.

The oxygen content of the composting pile can be effectively replenished using either pipe ventilation or regular turning. Pipeline ventilation replenishes oxygen well, but there is a high investment cost and production site must remain fixed. Compost turning is effective as well during the decomposition process, and it has the advantages of simple operation, flexible sites, and a low production cost; it can also improve the homogeneity of the compost material. Turning has been widely used in compost production, especially in windrow composting. According to previous studies, turning the composting pile once every 4–7 days is beneficial for compost fermentation (Zhang et al. 2016; Liu et al. 2018). However, the climate of the QTP is very different from that of other regions, which means that the optimal turning frequency for other regions may not be suitable for there. Based on this, it is necessary to determine the most appropriate turning frequency for compost fermentation in this region.

Microbial metabolism refers to a series of chemical processes in which microorganisms decompose substrates and absorb nutrients for growth and reproduction (Guo 2009). The abundance of functional genes, including the overall proportion of microbial functional genes (Wei et al. 2018; Zhang et al. 2020a; Li et al. 2021; Xu et al. 2021a) and the abundance of specific functional genes (Zhai et al. 2017; Weglarz et al. 2018; Jiang et al. 2020b), can be used to assess the metabolic capacity of microorganisms, and has been widely used in composting studies. Assessing the dynamics of enzymatic activity is also an effective way to deepen our understanding of microbial degradation of organic components and microbial metabolic function during composting (Zhang et al. 2021b). Extensive research has been conducted on enzyme activity during composting, including on effects of the carbon–nitrogen ratio (C/N) (Wu et al. 2017b), exogenous microorganisms (Li et al. 2020), and various additives (Awasthi et al. 2018, 2020b) on the enzymatic activity of composting. However, to the best of our knowledge, no studies have yet been conducted on the effects of turning frequency on microbial functional genes or enzymatic activity during composting. Therefore, studying this will enrich the knowledge in this field in regards to the microbial mechanisms of composting fermentation efficiency, as well as quality changes under different turning frequencies.

This study will explore the effects of turning frequency on the fermentation efficiency and microbial metabolic function of the windrow composting of sheep manure on the QTP. The main purposes of the experiment were as follows: to determine the effects of turning frequency on (1) the physicochemical properties and maturity of sheep manure compost; (2) the succession of microbial community structure and metabolic function; (3) the activity of functional enzymes.

## Material and methods

### Composting materials

Sheep manure (Euler sheep) and rape straw (forage rape, less than 5 mm in diameter) were obtained from Bakatai Farm (Qinghai Province, China). The rape straw was air-dried and crushed for use as a compost carbon nitrogen ratio and porosity regulator. Key physiochemical properties of raw materials are summarized in Table 1.

**Table1:** Basic physiochemical properties of composting raw materials

	Sheep manure	Rape straw	Mixture (4:1)
Organic matter content/%	74.2 ± 0.66	96.8 ± 0.58	78.6 ± 0.85
Total organic carbon content/%	43.1 ± 0.32	56.4 ± 0.51	45.8 ± 0.96
Total nitrogen content/%	2.43 ± 0.02	0.83 ± 0.01	2.11 ± 0.01
C/N	17.7 ± 0.18	67.9 ± 11.5	21.7 ± 0.78
pH value	7.75 ± 0.04	5.21 ± 0.05	7.69 ± 0.08
Electrical conductivity/mS cm^−1^	2.58 ± 0.02	0.62 ± 0.01	2.38 ± 0.02

### Experimental design and sampling

Sheep manure and rape straw were mixed evenly at a ratio of 4:1 dry weight (Table 1). Due to the low oxygen content of the QTP, the initial moisture content of this experiment was adjusted to 45%. Windrow composting was employed. The length, width, and height of the composting pile were 8 m, 2.5 m, and 1.3 m, respectively. The composting experiment was carried out at the Bakatai Farm, which is about 3300 m above sea level, and the experiment lasted for 36 d. Based on previous studies and the climatic characteristics of the QTP (Zhang et al. 2016; Liu et al. 2018; Guo et al. 2021), this experiment consisted of five treatments with different turning frequencies: turning once every 1 d (T1), 2 d (T2), 4 d (T3), 6 d (T4), and 8 d (T5). Three independent replicate experiments were performed for each treatment. All treatments were regularly replenished with tap water for the first 24 days of composting, up to the initial moisture content. Multi-point samples were gathered from different depths of the composting pile at 0, 4, 8, 12, 16, 20, 24, 30, and 36 d to improve representativeness and homogeneity. Then these samples were then evenly mixed and divided into two parts: one was immediately stored at 4 ℃ for analyzing the physicochemical parameters, and the other was stored at –20 ℃ for analyzing enzyme activity and DNA.

### Analytical methods

#### Determination of physicochemical parameters

The temperature was measured at 9:00 AM and 16:00 PM each day (Cai et al. 2022). Digital thermometers were evenly inserted into 8 positions on the upper (20 cm), middle (60 cm), and lower (100 cm) layers of the composting pile to calculate the average temperature of the 24 positions. The sample was placed in a blast dryer at 105 ℃ and dried for 24 h, and weight loss was determined to calculate the moisture content. The pH value and electrical conductivity (EC) value of the compost extract (1:10, w/v) were measured using a pH meter and a conductometer (Feng et al. 2021b). The total organic carbon (TOC) was measured using the method specified in the Chinese Ministry of Agriculture’s industry standard (NY 525-2021). The total nitrogen (TN) content was determined using an automatic Kjeldahl apparatus. The absorbance of the humic acid extracted from the sample was measured using an ultraviolet spectrophotometer at wavelengths of 465 nm and 665 nm, and the ratio of the two absorbance values was denoted as E_4_/E_6_ (Xu et al. 2021b). The germination index (GI) was determined and calculated as described by Arias et al. (2017). The humus content was determined as described by Li et al. (2021).

#### Determination of enzyme activity

The protease activity of compost was determined using ninhydrin colorimetry (Guan 1986); urease activity was determined using sodium phenolate sodium hypochlorite colorimetry (Wu et al. 2017b); cellulase activities were measured using the 3,5-dinitrosalicylic acid method (Guan 1986); β-glucosidase activity was determined using nitrophenol colorimetry (Eivazi & Tabatabai 1988); and the activities of peroxidase and polyphenol oxidase were determined using pyrogallol colorimetry (Guan 1986).

#### DNA extraction and PCR amplification

Genomic DNA was extracted from the sample (0.5 g) using a Fast DNA™ SPIN Kit for soil (MP Biomedicals, Irvine, CA, USA), according to the manufacturer’s instructions. The extracted genomic DNA was checked using 1% agarose gel electrophoresis. Polymerase chain reaction amplification (PCR) of bacterial and fungal DNA amplification were performed as described by Cong et al. (2020) and by Jiang et al. (2020a), respectively. The V3–V4 hypervariable region of the bacterial 16S rRNA gene was amplified using the primers 338F (ACTCCTACGGGAGGCAGCAG) and 806R (GGACTACHVGGGTWTCTAAT), and the universal primer pair ITS1-F (50-CTTGGTCATTTA-GAGGAAGTAA-30) and ITS2 (50-TGCGTTCTTCATC-GATGC-30) was used to target the ITS1 region of the fungal nuclear ribosomal repeat unit. Library construction and the Illumina MiSeq paired-end sequencing were performed by Beijing Allwegene Technology Co. Ltd. (Beijing, China) according to the manufacturer’s instructions.

### Statistical analysis

The averages and standard deviations of the physicochemical parameters and enzyme activity were calculated using SPSS 23.0 software. The bioinformatics tool, PICRUSt2 (Phylogenetic Investigation of Communities by Reconstruction of Unobserved States), was used to predict the function of the microbial community in the samples. Figures showing each indicator were produced using Origin 2021b. Correlations between the microbial communities and environmental factors were determined by redundancy analysis (RDA) using Canoco 5.

## Results and discussion

### Changes in physicochemcal indicators and maturity during composting

**Fig.1:**
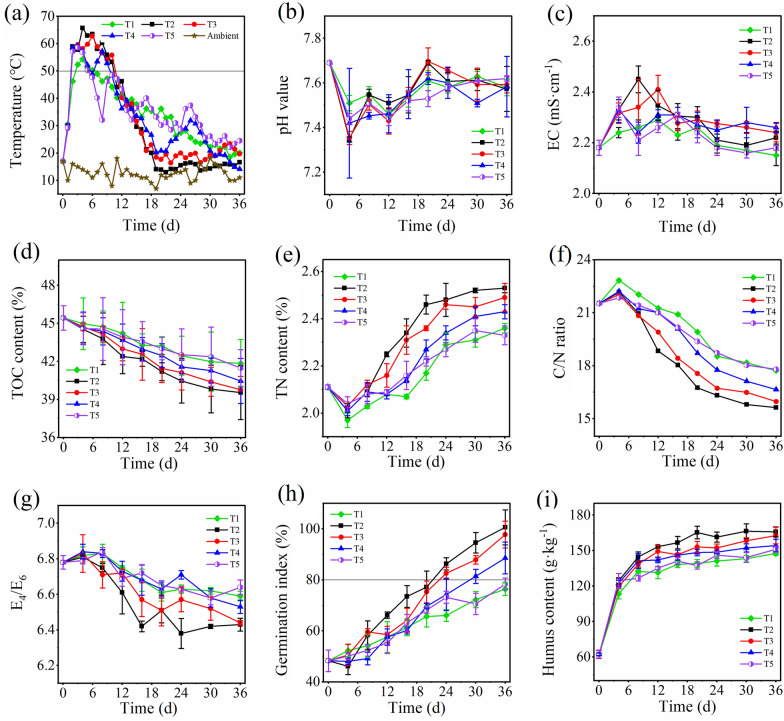
Changes in temperature (**a**), pH (**b**), EC (**c**), TOC content (**d**), TN content (**e**), C/N ratio (**f**), E_4_/E_6_ (**g**), germination index (**h**), and humus content (**i**) during composting

As shown in Fig. 1a, the temperature of the five treatments rose rapidly at the initial stage of composting and entered the high temperature stage (> 50 ℃) on the third day. The temperature of T2 and T3 during the 2nd to 10th day of composting was consistently over 50 ℃, with maximum temperatures of 65.7 ℃ and 62.8 ℃, respectively; this warming effect was better than other treatments at this stage. The temperature of T4 exceeded 50 ℃ on days 2–5 and 7–9, while T1 and T5 exceeded 50 ℃ on only four days; the maximum temperatures were 59.1 ℃, 54.3 ℃, and 58.6 ℃, respectively. This indicates that turning once every 2 days or 4 days is most beneficial to the increase of compost temperature. A proper turning frequency can effectively supplement the oxygen in the composting pile, which improves aerobic microbial activity and promotes the degradation of organic matter, which generate a lot of heat (Tan et al. 2022). A high turning frequency (T1) will lead to rapid temperature loss; unable to maintain a high temperature, it may end up reducing microbial metabolic activity. It is worth noting that the temperature of T4 and T5 rose rapidly on the 7th and 9th day, respectively, which was mainly due to the oxygen supplement after the turning, which caused the aerobic microorganisms in these two treatments to recover their metabolic activity. As the organic matter became consumed over time, the availability of compost material was restricted (Chen et al. 2018), and so the temperature of all treatments showed a downward trend as the compost matured. The temperature of T2 and T3 began to approach the ambient temperature and reached a thermal stable state on the 19th day of composting, and was significantly lower than that of the other treatments, indicating that T2 and T3 could reach the thermal stable state earlier, and T2 had the best effect.

As shown in Fig. 1b, the pH values of all treatments decreased in the first four days, which might have been related to the production and accumulation of large amounts of small molecular organic acids and CO_2_ (Li et al. 2012). Subsequently, due to organic acid degradation, NH_3_ volatilization, and the formation of a carbonate buffer system, the pH values of all treatments gradually increased and tended to be stable (Pan et al. 2019). By day 36 of composting, the pH values of T1–T5 were 7.58–7.62, and the differences were not significant. As shown in Fig. 1c, the EC value of all treatments increased during the early stage, primarily due to the rapid degradation and mineralization of organic matter into small molecular components (Pan et al. 2019). The EC values of T2 and T3 increased faster than that of the other treatments, which may have been caused by their faster fermentation speed. Subsequently, the EC values of all treatments decreased and then stabilized to about 2.2 mS·cm^−1^, which was related to the volatilization of organic acids and NH_4_^+^, the mineral salt precipitation, and the transformation of small molecular components into humus. At the end of composting, the EC values of T1–T5 were 2.15, 2.22, 2.24, 2.26, and 2.18 mS·cm^−1^, respectively. As shown in Fig. 1d, the TOC content of all treatments showed a decreasing trend in the whole composting process, mainly due to the release of CO_2_ from the TOC degradation in the compost materials (Zhang et al. 2020a). At the end of composting, the TOC content of T1–T5 was 41.8%, 39.6%, 39.8%, 40.5%, and 41.5%, respectively. The TOC content of T2 and T3 decreased faster than that of the other treatments, indicating that turning every 2 d or 4 d could improve the degradation rate. As shown in Fig. 1e, the TN content of all treatments showed a decreasing trend at the initial stage, mainly due to the rapid evaporation of NH_3_ produced by nitrogen metabolism at high temperature (Zhang et al. 2020a). Subsequently, the TN content of all treatments increased gradually and tended to be stable, which could be attributed to the concentration effect due to mass reduction with the rapid degradation of organic matter (Li et al. 2012). The TN content of T2 increased faster than that of other treatments, and T3 was second only to T2. By day 36 of composting, the TN of T1–T5 were 2.36%, 2.53%, 2.49%, 2.43%, and 2.33%, respectively.

The C/N ratio, E_4_/E_6_ ratio and germination index (GI) are commonly used to evaluate the toxicity degree and maturity of compost (Awasthi et al. 2020a). The C/N and E_4_/E_6_ ratio are negatively correlated with compost maturity, while GI is positively correlated with maturity (Cai et al. 2022). As shown in Fig. 1f, g, the C/N and E_4_/E_6_ ratio of all treatments were at a high level in the early stage, and then these two indicators showed a downward trend, gradually stabilizing in the late stage; this indicated that the maturing of the compost. At the end of composting, both indexes of T2 and T3 were lower than those of the other treatments, and that of T2 was slightly lower than that of T3, while the T4 and T5 indices were significantly higher than those of the other treatments. As shown in Fig. 1h, the GI of all treatments were lower than 50% in the first four days, which was associated with the production of large amounts of phenols, short-chain fatty acids, and NH_4_^+^ in the early stage (Meng et al. 2018). With the elimination of toxic substances, the GI of all treatments increased rapidly. It was reported that compost can be considered fully mature when GI is greater than 80% (Zhang et al. 2019, 2020b). According to this standard, T2 and T3 matured between the 20th and 24th days of composting, and T4 matured between the 30th and 36th days, while T1 and T5 remained immature. By day 36 of composting, the GI of T1–T5 reached 76.4%, 100.6%, 97.8%, 88.6%, and 78.4%, respectively. Humus is an organic modifier that can improve crop yield and soil fertility, which is a colloidal substance formed by decomposition and transformation of organic matter by microorganisms during composting (Li et al. 2021). As shown in Fig. 1i, the humus content of all treatments increased rapidly at the initial stage, and then gradually stabilized. By day 36 of composting, the humus content of T1–T5 were147.6, 165.8, 162.7, 154.3, and 151.2 g·kg^−1^, respectively. Figure 1a and Fig. 1f–i indicated that turning once every 2 d or 4 d can improve compost quality and shorten composting time. The compost quality of T2 was better than that of T3. The temperature performance and maturity of T1 and T5 were significantly worse than that of T2–T4. Therefore, turning once every 1 d or 8 d was not suitable for sheep manure composting on QTP.

Based on the physical and chemical indexes of the five treatments, T2, T3 and T4 were selected in this study to further analyze the changes of microbial metabolic function.

### Microbial diversity analysis and community structure

#### Changes in microbial alpha diversity

**Fig.2:**
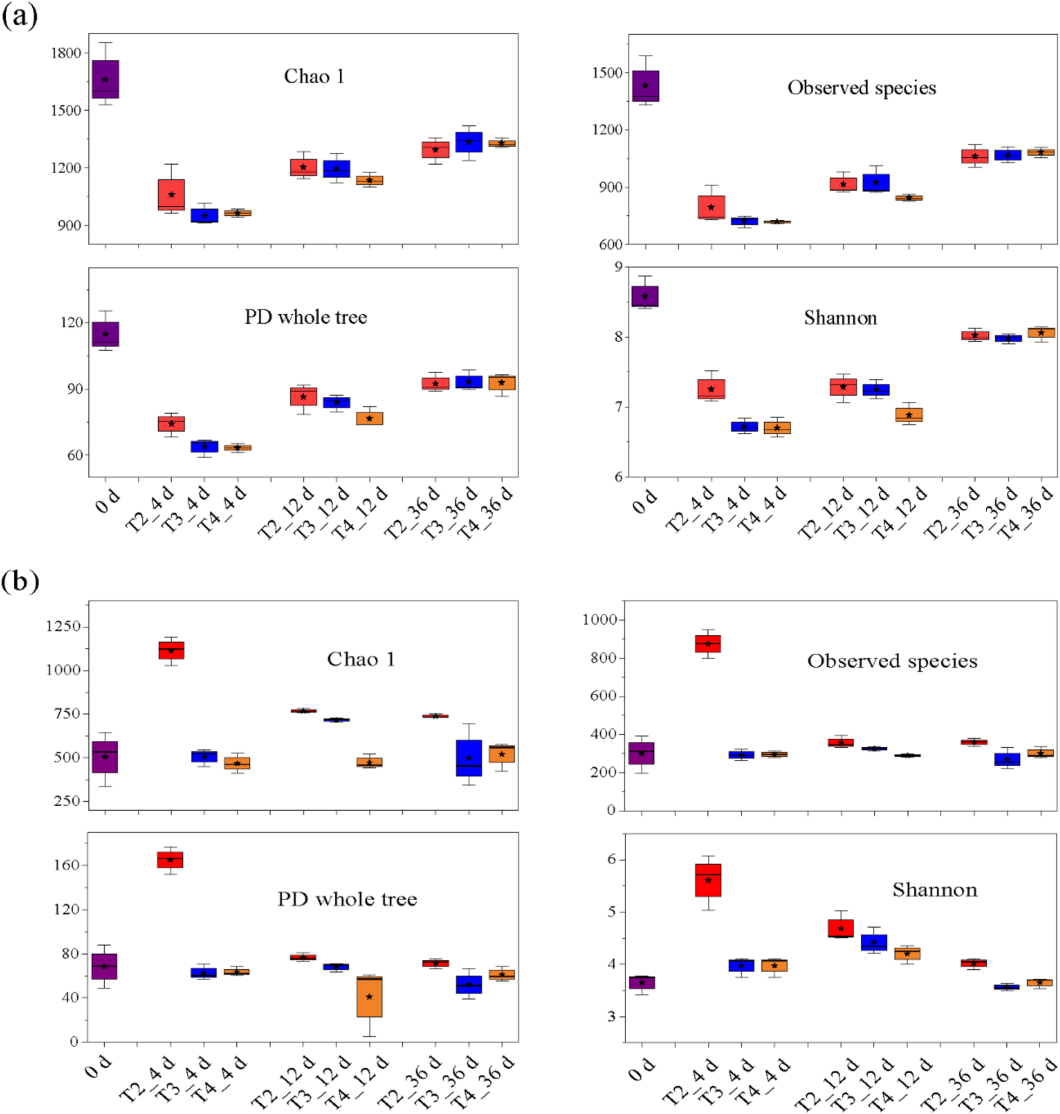
Changes in alpha diversity of bacteria (**a**) and fungi (**b**)

The Chao1 and the observed species indices were calculated to assess richness, and the phylogenetic diversity (PD) whole tree and Shannon indices were calculated to reflect the diversity (Cai et al. 2022; Zhou et al. 2022). As shown in Fig. 2a, the four alpha indices of bacteria in the three treatments decreased rapidly during the initial stage, and then showed an increasing trend. These four alpha indices of bacteria in T2 were higher than those in T3 and T4 on Day 4, while T4 was lower than T2 and T3 on Day 12. There were no significant differences among the three treatments on Day 36. As shown in Fig. 2b, the four alpha diversity indices of fungus in T2 increased rapidly during the first four days, then decreased rapidly and remained stable in the later composting period, while these indices did not change significantly in T3 and T4. The four fungal alpha diversity indices of T2 were significantly higher than those of T3 and T4 during the whole composting process. The Observed species and Shannon index of T3 were close to T4 on Day 4 and Day 36, and significantly higher on Day 12, while the Chao1 index was higher throughout the composting process. Figure 2 indicates that an appropriate increase in turning frequency can improve microbial richness and diversity during sheep manure composting on the QTP, which may due to its influence on the growth and development of aerobic microorganisms in the composting pile.

#### Changes in microbial community structure

**Fig.3:**
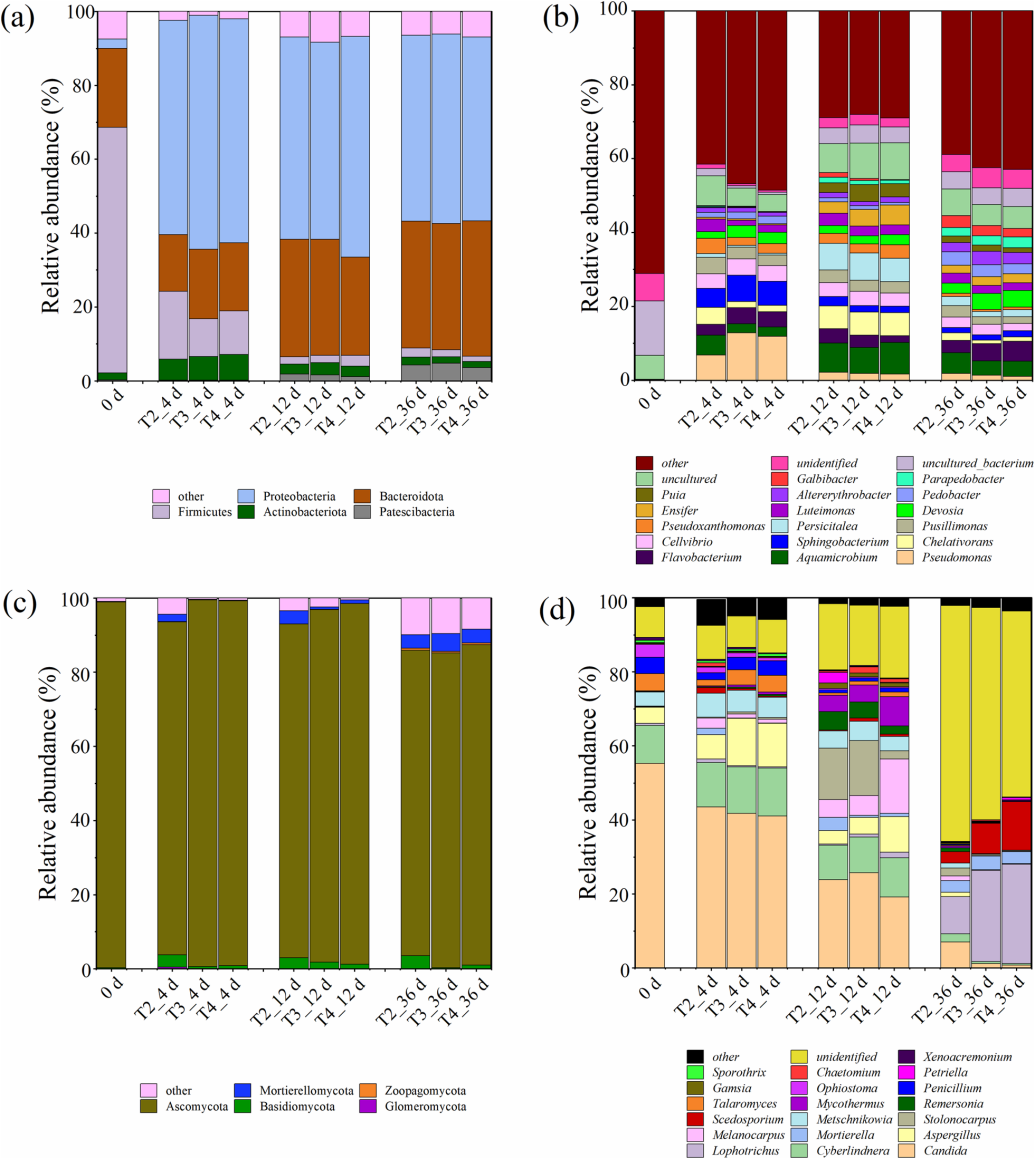
Changes in bacterial (**a**–**b**) and fungal (**c**–**d**) community structure

Figure 3a showed the bacterial community at the phylum level. Proteobacteria, Bacteroidetes, Firmicutes, Actinobacteria, and Patescibacteria were the dominant phyla during composting, these bacteria accounted for 91.7–99.0% of the total bacteria. According to previous research reports, these bacteria are the key ones that accelerates lignocellulose degradation, and the play an important role in the conversion and utilization of carbon and nitrogen (Ren et al. 2021; Zhou et al. 2021; Xu et al. 2022). During days 0–4, the relative abundance of Bacteroidetes in the three treatments was still higher than 15%, but Firmicutes decreased to less than 20%. At the same time, the abundance of Proteobacteria and Actinobacteria increased from 2.55% to more than 58%, and from 1.87% to more than 5.7%, respectively. The bacterial structure of the three treatments on Day 4 was different: the abundance of Firmicutes in T2 was higher than that in T3 and T4, while the abundance of Actinobacteria, Bacteroidetes, and Proteobacteria in T2 was lower than that in other treatments. From the 4th to the 12th day, the abundance of Proteobacteria in all treatments remained at 53.4–63.3%, while the abundance of Firmicutes and Actinobacteria decreased rapidly and the abundance of Bacteroidetes showed an upward trend. Subsequently, the bacterial community structure at the phylum level gradually stabilized, and the total abundance of Proteobacteria and Bacteroidetes exceeded 84% at this stage; this trend was similar to previous research (Cai et al. 2023b). In addition, the relative abundance of Patescibacteria showed an increasing trend. Figure 3b shows the variations in the bacterial community structure at the genus level. The dominant genus of bacteria in compost materials were uncultured and unidentified bacteria. With the increase of compost temperature, the abundance of, for example, *Pseudomonas*, *Aquamicrobium*, *Flavobacterium*, *Sphingobacterium*, *Cellvibrio*, and *Pusillimonas*, increased rapidly. It has been reported that these bacteria were prone to appear in immature piles, and their relative abundance was positively correlated with EC value and negatively correlated with pH value and moisture content (Cai et al. 2023b). Subsequently, the abundance of *Aquamicrobium*, *Chelativorans*, *Persicitalea*, *Ensifer*, and *Puia* increased from the 4th to 12th day, and then decreased from the 12th to 36th day. At the same time, *Devosia*, *Pedobacter*, *Altererythrobacter*, *Parapedobacter*, and *Galbibacter* showed an increasing trend during the late stage; these bacteria are closely related to compost maturity and lignocellulosic degradation (Wang et al. 2021; Zhang et al. 2022; Cai et al. 2023b).

As shown in Fig. 3c, Ascomycota was the most important fungus phyla in the whole composting process (82.4–98.9%). Ascomycota has good high-temperature adaptability and can utilize a variety of carbon sources, and is considered the dominant phylum in lignocellulosic compost ecosystems (Zhang et al. 2011, 2021a; Cai et al. 2022). The relative abundance of Ascomycota in T2 was slightly lower than that in T3 and T4 during composting, but the fungal richness in T2 was significantly higher than that in T3 and T4 (Fig. 2). Therefore, the absolute number of Ascomycetes in T2 may have been higher than that in T3 and T4. The relative abundance of Basidiomycota in T2 remained at 2.93–3.51% during the whole composting process, while in T3 and T4 it was always less than 2%. Basidiomycota have been reported to produce special stress-resistant structures that resist adverse composting environments and play an important role as decomposers during composting (Jiang et al. 2020c). The relative abundance of Mortierellomycota in all the treatments showed an upward trend during composting. Figure 3d shows that the most abundant genera in the raw compost materials were *Candida*, *Cyberlindnera*, *Aspergillus*, *Metschnikowia*, *Talaromyces*, *Penicillium*, and *Ophiostoma*, and their relative abundance was more than 3%. During the high temperature period, the relative abundance of *Candida*, *Penicillium*, *Talaromyces*, and *Ophiostoma* in all treatments showed a downward trend, while that of *Cyberlindera*, *Aspergillus*, and *Metschnikowia* showed an upward trend. The total abundance of *Candida*, *Cyberlindera*, and *Aspergillus* of the three treatments was more than 60% on Day 4, indicating that three fungi were the dominant fungi in the thermophilic stage. *Melanocarpus*, *Mycothermus*, and *Metschnikowia* were also the main fungi in the early stage. It has been reported that these fungi can catalyze the degradation of organic components by secreting different enzymes during the thermophilic phase of composting (Duan et al. 2021; Feng et al. 2021a; Cai et al. 2022). On the 4th to 12th day, the relative abundance of *Candida*, *Aspergillus*, *Cyberlindnera*, *Metschnikowia*, *Penicillium*, and *Talaromyces* declined, while the abundance of *Melanocarpus*, *Stolonocarpus*, *Remersonia*, and *Mycothermus* increased rapidly. On Day 12, the abundance of *Candida*, *Stolonocarpus*, *Metschnikowia*, and *Remersonia* in T2 and T3 was higher than that in T4, while that in *Aspergillus*, *Melanocarpus*, and *Mycothermus* was lower than that in T4. In the mature stage of composting, the abundance of *Lophototrichus*, *Mortierella*, and *Scedosporium* increased rapidly, while that of other fungi decreased. At the end of composting, the relative abundance of *Candida* in T2 was higher than that in T3 and T4, while *Lophotrichus* and *Scedosporium* were the opposite, indicating that an appropriate increase in the turning frequency can change the fungal structure in compost.

### Microbial function prediction during composting

#### Bacterial community function prediction during composting

**Fig. 4:**
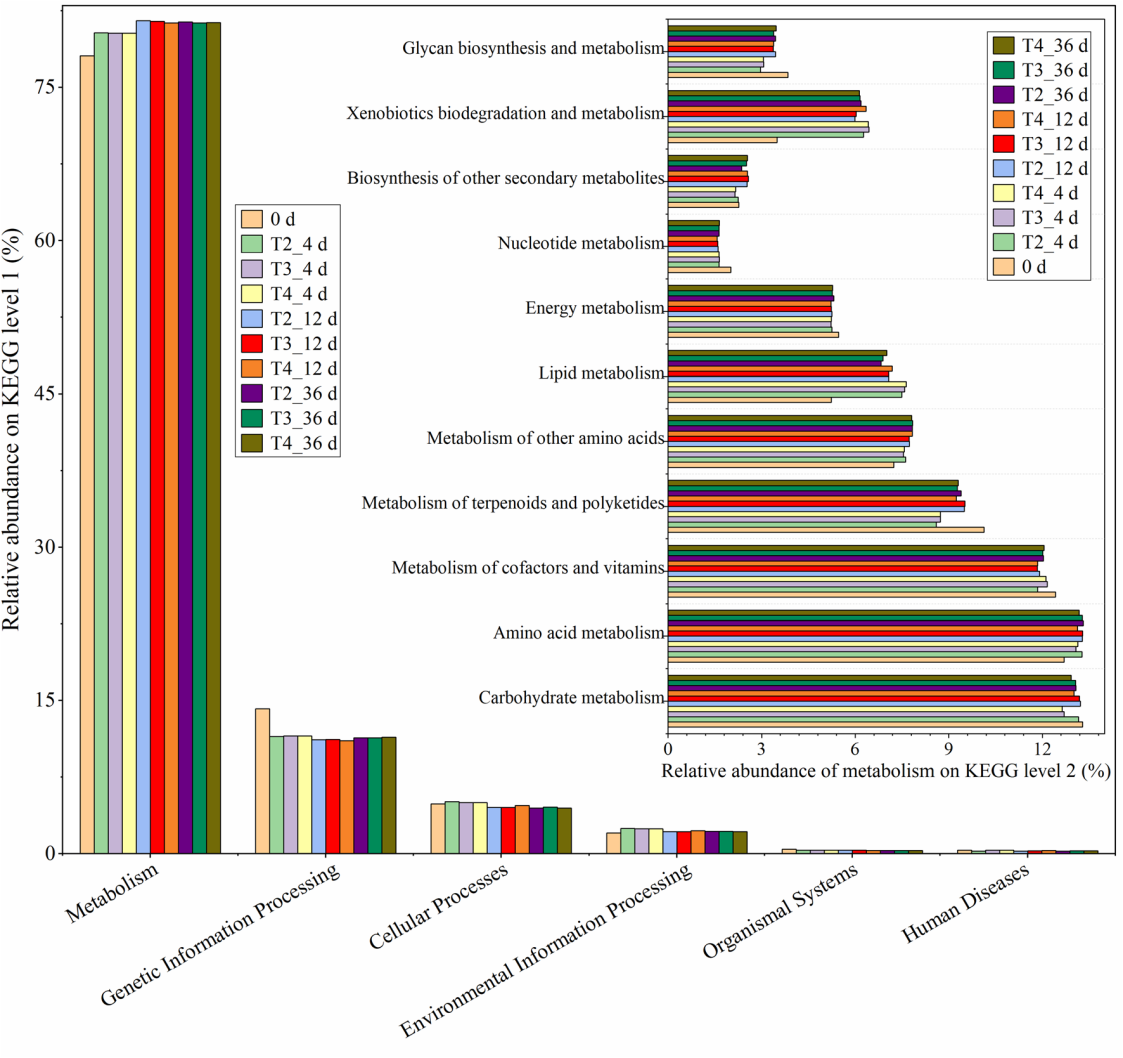
Prediction of the abundance of bacterial metabolic function based on PICRUSt2

PICRUSt2 software was used to predict the bacterial community function of sheep manure composting, and the result on KEGG level 1 is shown in Fig. 4a. It can be divided into six functional groups, including metabolism, genetic information processing, cellular processes, environmental information processing, organismal systems, and human diseases. Metabolism accounted for 78.1–81.5% during composting, which indicates the importance of this function. The relative abundance of genetic information processing was second only to that of metabolism, and its value was 11.1–14.2% during composting. The result on KEGG level 2 of metabolism is shown in Fig. 4b, which indicated that there were 11 pathways for metabolism. The main metabolic pathways were carbohydrate, amino acid, and cofactors and vitamins metabolism, and their relative abundance more than 10% during composting. Similar results have been reported elsewhere (Zhang et al. 2020a; Li et al. 2021). It has been reported that carbohydrate metabolism is closely related to the biodegradation of organic component such as lignocellulose during composting (Wei et al. 2018), and that the more gene sequences involved in amino acid metabolism during composting, the better the production of amino acids and humus (Wu et al. 2017a; Zhang et al. 2020a). The abundance of functional genes in carbohydrate metabolism and amino acid metabolism in T2 was higher than that in T3 and T4, which may have led to the superior lignocellulosic degradation and humus synthesis in T2. In addition, the relative abundance of functional genes of energy metabolism in T2 was higher than that in T3 and T4. The abundance of lipid metabolism, xenobiotics biodegradation and metabolism, and metabolism of terpenoids and polyketides in all treatments exceeded 5% during composting, which indicated that these metabolic pathways also played an important role.

#### Prediction of gene abundance involved in fungal metabolic

**Fig. 5:**
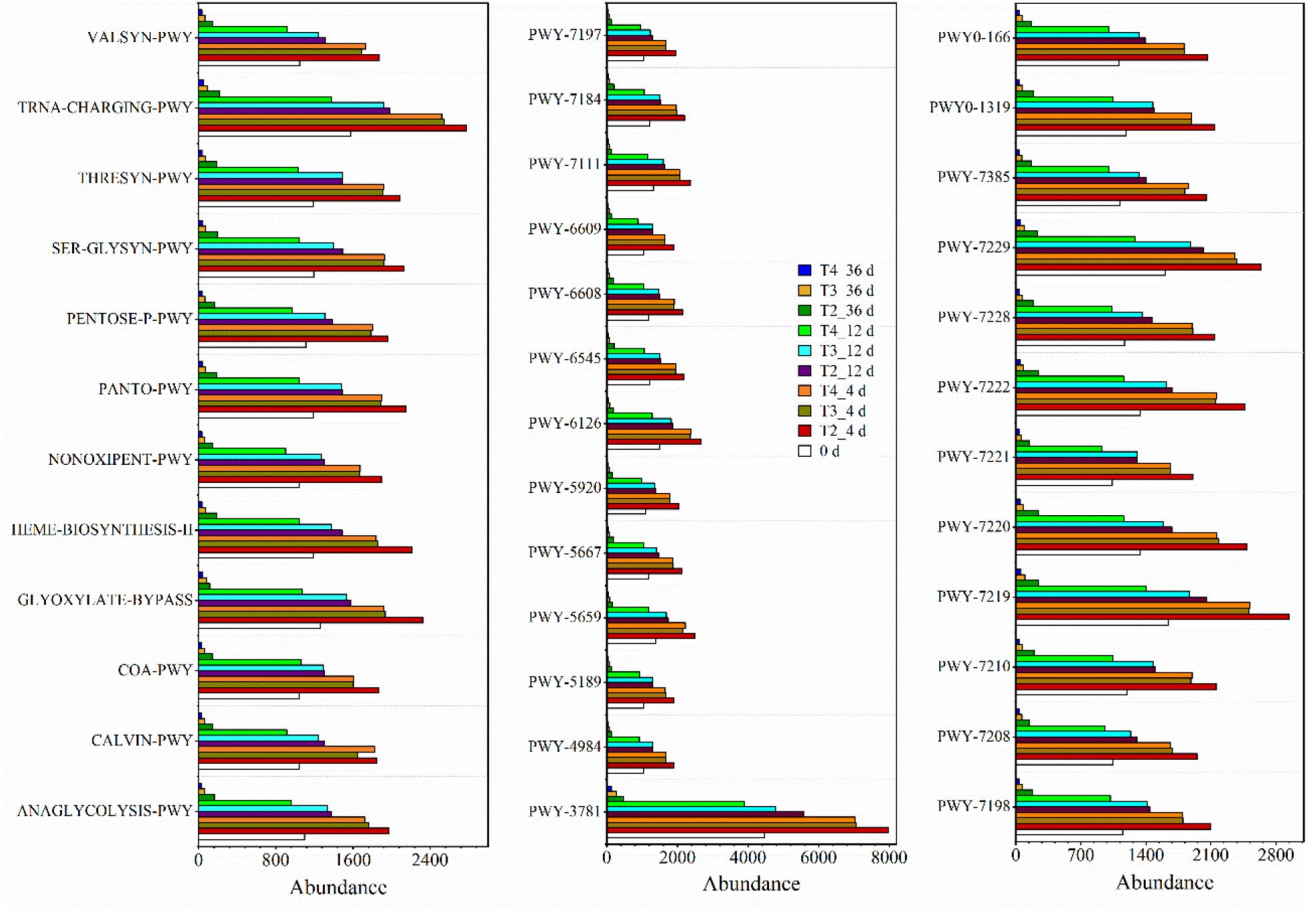
Prediction of the abundance of fungal metabolic pathways based on PICRUSt2

The predicted results of the abundance of genes related to fungal metabolic pathways are shown in Fig. 5. A total of 37 metabolic pathways with high abundance were selected. Among these pathways, aerobic respiration I (PXY-3781) had the highest gene abundance, with its functional gene abundance exceeding 7000 on Day 4, while the other pathways had abundance between 1600 and 3000. Aerobic respiration I is an important pathway of microbial energy metabolism, which is itself an important process for converting energy generated by organic degradation into adenosine triphosphate (ATP), so this function plays an important role in fungal metabolism (Cai et al. 2023a). Except for aerobic respiration I, organic component metabolism was one of the main fungal metabolic pathways during composting, and these pathways were mainly involved in carbohydrate, protein and lipid metabolism, including glycolysis (ANAGLYCOLYSIS-PWY), the pentose phosphate pathway (NONOX-IPENT-PWY, PENTOSE-P-PWY), the glyoxylate cycle (GLYOXYLATE-BYPASS), urea cycle (PWY-4984), and amino acid synthesis (SER-GLYSYN-PWY, THRESYN-PWY, VALSYN-PWY, etc.). In addition, nucleotide metabolism was also the main metabolic pathway, including PWY-6608, 6545, 7219-7222 and 7229. The 37 metabolic pathways showed the same trend in the whole composting process, increasing rapidly at the initial stage and then decreasing. The abundance of functional genes related to these metabolic pathways in T2 was higher than that in other treatments throughout the composting process. On Day 4, the abundance of these functional genes showed no significant difference between T3 and T4, while T4 was lower than T3 on Day 12 and Day 36. This indicated that appropriately increasing the turning frequency could improve the fungal metabolic capacity, and that the metabolic capacity of fungi was improved best by turning once every 2 days.

### Changes in enzyme activity

**Fig. 6:**
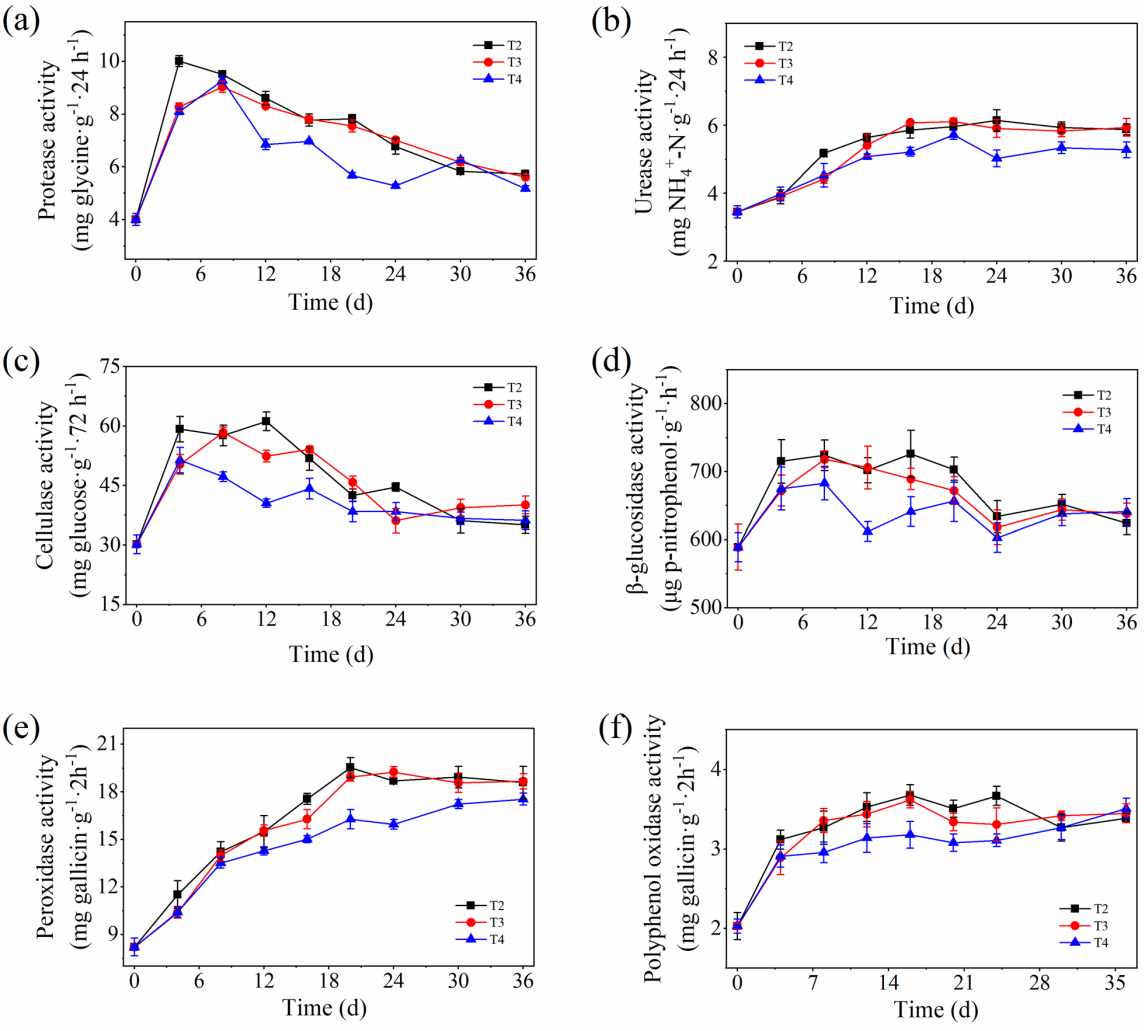
Changes in protease (**a**), urease (**b**), cellulase (**c**), β-glucosidase (**d**), peroxidase (**e**), and polyphenol oxidase (**f**) activity during composting

Protease and urease are important participants in nitrogen metabolism, among which protease mediates the first step of mineralization and is often the rate-limiting step in the nitrogen cycle, while urease can catalyze the hydrolysis of urea to produce carbonic acid and ammonia (Ma et al. 2019; Liang et al. 2020; Cai et al. 2022). As shown in Fig. 6a, the protease activity of all treatments increased rapidly in the early stage and maintained high activity at this stage, in which the activity value of T2 was higher than that of other treatments. During the same period, the urease activity of all treatments was at a low level and showed a slow increasing trend (Fig. 6b), which was similar to some previous reports, indicating that urease is sensitive to high temperatures (Cai et al. 2022; Duan et al. 2022). Subsequently, the protease activity of all treatments decreased rapidly, and was then maintained at a low level, while the urease activity increased continuously and then stabilized. The activity of these two enzymes in T4 was lower than that of T2 and T3 in the middle and late stages of composting.

Cellulase and β-glucosidase can catalyze the decomposition of cellulose into oligosaccharides or monosaccharides, which are beneficial for the utilization of microorganisms (Jiang et al. 2020d; Li et al. 2020). As shown in Fig. 6c, d, the activities of cellulase and β-glucosidase in all treatments increased rapidly in the early period, which effectively promoted the degradation of cellulose in the compost. Subsequently, the activities of these two enzymes in T2 and T3 remained at a high level and then decreased to a stable state, while T4 began to decrease, gradually stabilizing after the 4th day of composting. The activity of these two enzymes in T2 was higher than that in T3 and T4 at the early stage, while T4 was significantly lower than that in other treatments.

Peroxidase can oxidize the lignin polymer, while polyphenol oxidase plays an important role in the conversion of aromatic compounds; both enzymes can promote the humus formation (Ma et al. 2019; Cai et al. 2022). As shown in Fig. 6e, f, the activities of peroxidase and polyphenol oxidase in all treatments showed an increasing trend in the early and middle stages. The activities of these two enzymes in T4 were significantly lower than that in T2 and T3 in the early and middle stages. During the late period of composting, the activities of peroxidase and polyphenol oxidase in T2 and T3 remained stable, while those of T4 showed a slow increasing trend. However, the activities of these two enzymes were still lower than those of T2 and T3.

Figure 6 shows that turning once every 2 days is conducive to improving the activity of multiple functional enzymes during composting on the QTP, especially in the rapid fermentation stage. This is because increasing the turning frequency can increase the oxygen supplement, thus, improving the activity of aerobic microorganisms and facilitating the secretion of extracellular enzymes by these microorganisms; this promotes compost fermentation and improves the maturity of compost products.

### Correlation between physiochemical properties and microbial communities

**Fig. 7:**
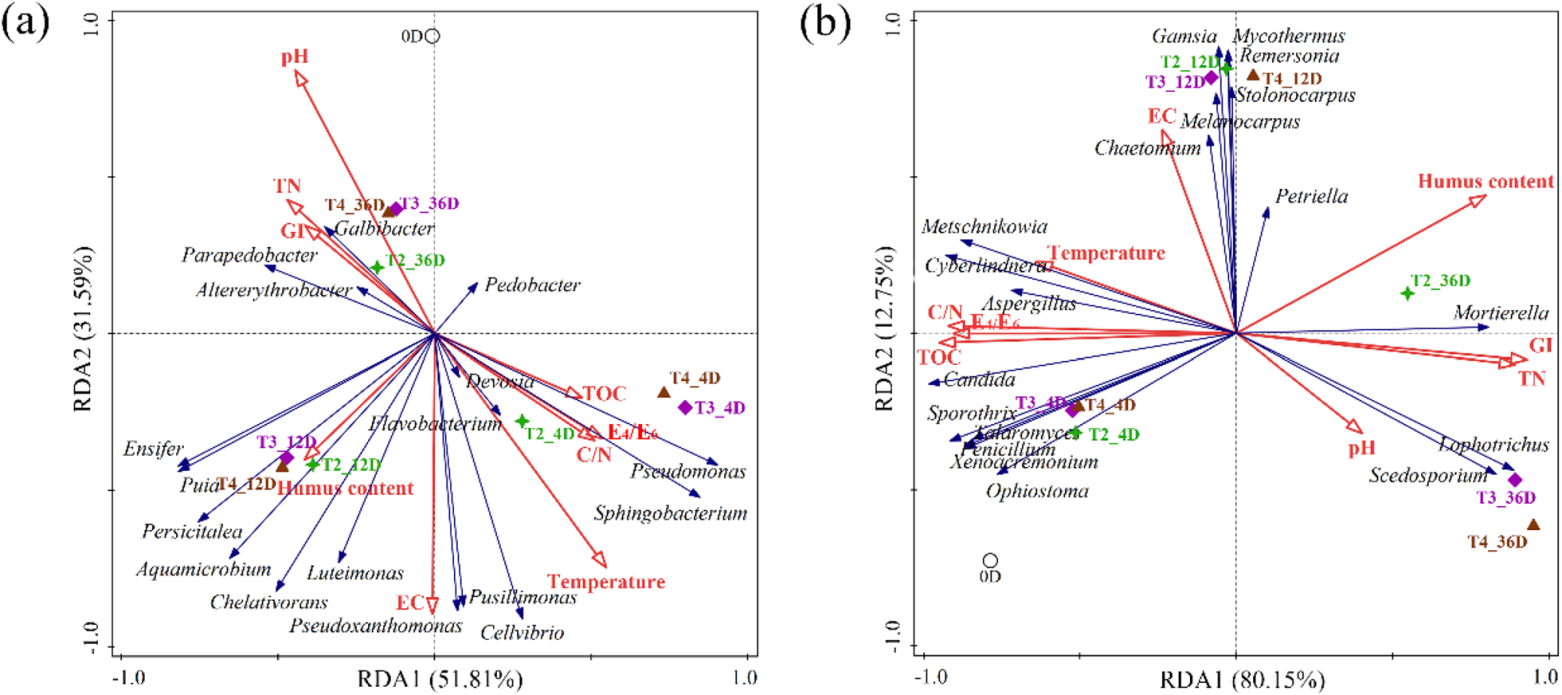
Correlation between physiochemical properties and microbial communities (**a** bacteria; **b** fungus)

The results of the RDA between the bacterial community and the environmental factors are presented in Fig. 7a. The first and second axes explain 51.81% and 32.59% of the variance, respectively. Among all environmental factors, temperature (34.0%, *p* = 0.006) was the greatest contributor, followed by EC (26.3%, *p* = 0.032) and the E_4_/E_6_ ratio (13.5%, *p* = 0.012). The correlation between the remaining environmental factors and the bacterial community did not reach statistical significance (*p* > 0.05). However, this does not mean that these factors did not affect the bacterial community. Figure 7a also shows that *Sphingobacterium* and *Pseudomonas* were positively correlated with TOC content, E_4_/E_6_ and C/N, and negatively correlated with TN content and GI, while *Galbibacter* and *Parapedobacter* were the opposite. *Pseudoxanthomonas* and *Pusillimonas* were positively correlated with EC, while *Persicitalea* and *Aquamicrobium* were positively correlated with humus content.

The results of the RDA between the fungal community and the environmental factors are presented in Fig. 7b. The first and second axes explain 80.15% and 12.75% of the variance, respectively. Among all environmental factors, the TOC content (72.6%, *p* = 0.002) was the greatest contributor, followed by temperature (5.1%, *p* = 0.048). Figure 7b also shows that *Metschnikowia*, *Cyberlindnera* and *Aspergillus* were positively correlated with temperature, but negatively correlated with GI and TN content; *Candida* was positively correlated with TOC, C/N and E_4_/E_6_, but negatively correlated with GI and TN content. *Lophotrichus* and *Scedosporium* were positively correlated with pH, while *Chaetomium*, *Melanocarpus* and *Gamsia* were positively correlated with EC.

These results indicate that temperature can affect the bacterial and fungal community structure in compost. Appropriately increasing the turning frequency can promote an increase in composting temperature and improve microbial community structure, thereby improving the quality of compost on the QTP.

## Conclusion

Turning once every day or once every 8 days will result in the inability of sheep manure compost to reach maturity on the Qinghai–Tibet Plateau, while turning once every 2 to 6 days is conducive to compost maturity and improving composting efficiency. The composting efficiency and compost product maturity are highest when turning once every two days. Turning once every 2 days can increase the abundance of functional genes related to carbohydrate metabolism, protein metabolism, and energy metabolism, and improve the activity of enzymes such as protease, cellulase and peroxidase. Based on this, it is recommended that the turning frequency for windrow composting of sheep manure on the Qinghai–Tibet Plateau should be once every 2 days. This study provides theoretical basis and technical support for the efficient production of sheep manure compost on the Qinghai–Tibet Plateau.

## Data Availability

Data are available on request to the authors.

## Abbreviations

QTP: Qinghai–Tibet Plateau
C/N: Carbon–nitrogen ratio
EC: Electrical conductivity
TOC: Total organic carbon
TN: Total nitrogen
GI: Germination index
PCR: Polymerase chain reaction
RDA: Redundancy analysis
